# Supplementary material for: Disentangling acute motor deficits and adaptive responses evoked by the loss of cerebellar output
Source: eLife. 2025 Jun 25;14:RP105152. doi: 10.7554/eLife.105152 (PMC12194117; doi:10.7554/eLife.105152)
Supplement: Supplementary file 1. [file elife-105152-supp1.docx]

Supplementary file 1: Mean success rate across sessions per monkey.

| **Monkey** | **Success rate (Control)** | **Success rate (Cerebellar block)** |
| --- | --- | --- |
| Monkey S | 84.9% CI [82.2, 87.5] | 66.8% CI [63.4, 70.3] |
| Monkey C | 89.9% CI [88.8, 90.9] | 82.3% CI [79.8, 84.7] |
| Monkey M | 80.4% CI [79.1, 81.6] | 66.1% CI [63.4, 68.7] |
| Monkey P | 90.4% CI [89.3, 91.5] | 82.4% CI [80.0, 84.6] |
