# Supplementary material for: Disentangling acute motor deficits and adaptive responses evoked by the loss of cerebellar output
Source: eLife. 2025 Jun 25;14:RP105152. doi: 10.7554/eLife.105152 (PMC12194117; doi:10.7554/eLife.105152)
Supplement: Supplementary file 3. — (a) ANOVA marginal tests for the effect of target group (targets 1–4 vs. targets 5–8) on peak hand velocity during control (DF: degrees of freedom). Movements exhibited no significant difference in peak hand velocity during the outward reaching (targets 1–4) vs. retrieval (targets 5–8) movements during the control condition. The peak hand velocity was modeled using a linear mixed-effects model, with the target group (T1-4 vs. T5-8) as a fixed effect and random intercepts and slopes for the target group within each subject (i.e. monkey). The median peak hand velocity during the control trials was computed for each session. The input to the model was the target group-wise values computed from all sessions pooled across all four monkeys. (b) Mean peak hand velocity for outward reaching (targets 1–4) vs. inward reaching (targets 5–8) movements during control across all sessions per monkey. For each session, the median peak hand velocity was computed across all the control trials per target group. Then, the mean and confidence intervals of the mean were computed from the per-session data for each monkey. [file elife-105152-supp3.docx]

Supplementary file 3a: ANOVA marginal tests for the effect of target group (targets 1-4 vs. targets 5-8) on peak hand velocity during control. (DF: degrees of freedom)

| **Model: Peak velocity ~ Target group + (1 + Target group \| Subject)** | | | | |
| --- | --- | --- | --- | --- |
| **Term** | **F-Statistic** | **DF1** | **DF2** | **p-value** |
| Intercept | 114.80 | 1 | 774 | < 0.001 |
| Target group | 0.02 | 7 | 774 | 0.889 |

Description: Movements exhibited no significant difference in peak hand velocity during the outward reaching (targets 1-4) vs. retrieval (targets 5-8) movements during the control condition. The peak hand velocity was modeled using a linear mixed-effects model, with the target group (T1-4 vs. T5-8) as a fixed effect and random intercepts and slopes for the target group within each subject (i.e. monkey). The median peak hand velocity during the control trials was computed for each session. The input to the model was the target group-wise values computed from all sessions pooled across all four monkeys.

Supplementary file 3b: Mean peak hand velocity for outward reaching (targets 1-4) vs. inward reaching (targets 5-8) movements during control across all sessions per monkey. For each session, the median peak hand velocity was computed across all the control trials per target group. Then, the mean and confidence intervals of the mean were computed from the per-session data for each monkey.

| **Monkey** | **Targets 1-4**  **(mean ± CI, cm/s)** | **Targets 5-8**  **(mean ± CI, cm/s)** |
| --- | --- | --- |
| Monkey S | 18.2 [17.3, 19.0] | 18.4 [17.5, 19.1] |
| Monkey C | 12.5 [11.4, 13.0] | 11.5 [10.9, 12.8] |
| Monkey M | 14.6 [14.3, 15.1] | 15.1 [14.8 15.5] |
| Monkey P | 11.2 [10.8, 11.6] | 12.3 [11.9, 12.7] |
