# Supplementary material for: Disentangling acute motor deficits and adaptive responses evoked by the loss of cerebellar output
Source: eLife. 2025 Jun 25;14:RP105152. doi: 10.7554/eLife.105152 (PMC12194117; doi:10.7554/eLife.105152)
Supplement: Supplementary file 4. — Movements exhibited a significant reduction in shoulder muscle torque impulse during the cerebellar block in a target-dependent manner. The torque impulse was computed by integrating the torque profile during the positive acceleration phase of the movement. The change in muscle torque impulse was modeled using a linear mixed-effects model, with target as a fixed effect and random intercepts and slopes for target within each subject (i.e. monkey). For each session, the target-wise change in the median muscle torque impulse during the cerebellar block trials was computed relative to that of control trials. The input to the model was the target-wise values computed from all sessions pooled across all four monkeys. The significant effect of target direction on the change in muscle torque impulse can be interpreted as analogous to the interaction between cerebellar block and target direction on the actual peak velocities. [file elife-105152-supp4.docx]

Supplementary file 4: ANOVA marginal tests for the effect of target direction on the change in shoulder muscle torque impulse due to cerebellar block relative to control. (DF: degrees of freedom)

| **Model: Shoulder muscle torque change ~ Target + (1 + Target \| Subject)** | | | | |
| --- | --- | --- | --- | --- |
| **Term** | **F-Statistic** | **DF1** | **DF2** | **p-value** |
| Intercept | 7.22 | 1 | 774 | 0.01 |
| Target | 12.55 | 7 | 774 | < 0.001 |

**Description:** Movements exhibited a significant reduction in shoulder muscle torque impulse during the cerebellar block in a target-dependent manner. The torque impulse was computed by integrating the torque profile during the positive acceleration phase of the movement. The change in muscle torque impulse was modeled using a linear mixed-effects model, with target as a fixed effect and random intercepts and slopes for target within each subject (i.e. monkey). For each session, the target-wise change in the median muscle torque impulse during the cerebellar block trials was computed relative to that of control trials. The input to the model was the target-wise values computed from all sessions pooled across all four monkeys. The significant effect of target direction on the change in muscle torque impulse can be interpreted as analogous to the interaction between cerebellar block and target direction on the actual peak velocities.
