# Supplementary material for: Disentangling acute motor deficits and adaptive responses evoked by the loss of cerebellar output
Source: eLife. 2025 Jun 25;14:RP105152. doi: 10.7554/eLife.105152 (PMC12194117; doi:10.7554/eLife.105152)
Supplement: Supplementary file 5. — Movements exhibited a significant reduction in elbow muscle torque impulse during the cerebellar block in a target-dependent manner. The torque impulse was computed by integrating the torque profile during the positive acceleration phase of the movement. The change in muscle torque impulse was modeled using a linear mixed-effects model, with target as a fixed effect and random intercepts and slopes for target within each subject (i.e. monkey). For each session, the target-wise change in the median muscle torque impulse during the cerebellar block trials was computed relative to that of control trials. The input to the model was the target-wise values computed from all sessions pooled across all four monkeys. [file elife-105152-supp5.docx]

Supplementary file 5: ANOVA marginal tests for the effect of target direction on the change in elbow muscle torque impulse due to cerebellar block relative to control. (DF: degrees of freedom)

| **Model: Elbow muscle torque change ~ Target + (1 + Target \| Subject)** | | | | |
| --- | --- | --- | --- | --- |
| **Term** | **F-Statistic** | **DF1** | **DF2** | **p-value** |
| Intercept | 0.46 | 1 | 774 | 0.50 |
| Target | 0.66 | 7 | 774 | 0.70 |

**Description:** Movements exhibited a significant reduction in elbow muscle torque impulse during the cerebellar block in a target-dependent manner. The torque impulse was computed by integrating the torque profile during the positive acceleration phase of the movement. The change in muscle torque impulse was modeled using a linear mixed-effects model, with target as a fixed effect and random intercepts and slopes for target within each subject (i.e. monkey). For each session, the target-wise change in the median muscle torque impulse during the cerebellar block trials was computed relative to that of control trials. The input to the model was the target-wise values computed from all sessions pooled across all four monkeys.
