# Supplementary material for: Disentangling acute motor deficits and adaptive responses evoked by the loss of cerebellar output
Source: eLife. 2025 Jun 25;14:RP105152. doi: 10.7554/eLife.105152 (PMC12194117; doi:10.7554/eLife.105152)
Supplement: Supplementary file 6. — The evolution of peak hand velocities during control vs. cerebellar block was analyzed by preserving the order of presentation of the target in each block of trials (i.e. trial sequence). For each monkey, the peak hand velocities were normalized to the median peak velocity of the early trials 1–2 in the control blocks. The normalized peak velocities were then modeled using a linear mixed-effects model, with trial type (control/cerebellar block) and trial sequence (1-20) as fixed effects and random intercepts and slopes for trial type and trial sequence within each subject (i.e. monkey). [file elife-105152-supp6.docx]

Supplementary file 6: ANOVA marginal tests for the effect of trial sequence and trial type (control/cerebellar block) on the peak hand velocity relative to 1^st^ 2 trials in control for movements to target 1. (DF: degrees of freedom)

| **Model: Peak velocity (%) ~ Trial type x Trial sequence + (1 + Trial type x Trial sequence \| Subject)** | | | | |
| --- | --- | --- | --- | --- |
| **Term** | **F-Statistic** | **DF1** | **DF2** | **p-value** |
| Intercept | 7472.51 | 1 | 293 | < 0.001 |
| Trial type | 8.35 | 1 | 293 | 0.004 |
| Trial sequence | 0.03 | 1 | 293 | 0.957 |
| Trial type : Trial sequence | 0.16 | 1 | 293 | 0.693 |

**Description:** The evolution of peak hand velocities during control vs. cerebellar block was analyzed by preserving the order of presentation of the target in each block of trials (i.e., trial sequence). For each monkey, the peak hand velocities were normalized to the median peak velocity of the early trials 1-2 in the control blocks. The normalized peak velocities were then modeled using a linear mixed-effects model, with trial type (control/cerebellar block) and trial sequence (1-20) as fixed effects and random intercepts and slopes for trial type and trial sequence within each subject (i.e. monkey).
