# Supplementary material for: Disentangling acute motor deficits and adaptive responses evoked by the loss of cerebellar output
Source: eLife. 2025 Jun 25;14:RP105152. doi: 10.7554/eLife.105152 (PMC12194117; doi:10.7554/eLife.105152)
Supplement: Supplementary file 8. — The evolution of decomposition index (measured as the fraction of time during a movement when either, but not both, of the shoulder or the elbow joint velocity was less than 20°/s) during control vs. cerebellar block was analyzed by preserving the order of presentation of the target in each block of trials (i.e. trial sequence). For each monkey, the decomposition indices were normalized to the median decomposition index of the early trials 1–2 in the control blocks. The normalized decomposition indices were then modeled using a linear mixed-effects model, with trial type (control/cerebellar block) and trial sequence (1-20) as fixed effects and random intercepts and slopes for trial type and trial sequence within each subject (i.e. monkey). [file elife-105152-supp8.docx]

Supplementary table 8: ANOVA marginal tests for the effect of trial sequence and trial type (control/cerebellar block) on movement decomposition relative to 1^st^ 2 trials in control for movements to targets 2-4. (DF: degrees of freedom)

| **Model: Decomposition index (%) ~ Trial type x Trial sequence + (1 + Trial type x Trial sequence \| Subject)** | | | | |
| --- | --- | --- | --- | --- |
| **Term** | **F-Statistic** | **DF1** | **DF2** | **p-value** |
| Intercept | 884.41 | 1 | 1021 | < 0.001 |
| Trial type | 52.97 | 1 | 1021 | < 0.001 |
| Trial sequence | 2.08 | 1 | 1021 | 0.149 |
| Trial type : Trial sequence | 0.63 | 1 | 1021 | 0.426 |

**Description:** The evolution of decomposition index (measured as the fraction of time during a movement when either, but not both, of the shoulder or the elbow joint velocity was less than 20°/s) during control vs. cerebellar block was analyzed by preserving the order of presentation of the target in each block of trials (i.e., trial sequence). For each monkey, the decomposition indices were normalized to the median decomposition index of the early trials 1-2 in the control blocks. The normalized decomposition indices were then modeled using a linear mixed-effects model, with trial type (control/cerebellar block) and trial sequence (1-20) as fixed effects and random intercepts and slopes for trial type and trial sequence within each subject (i.e. monkey).
